# Supplementary material for: Learning curve and surgical outcome of robotic assisted colorectal surgery with ERAS program
Source: Sci Rep. 2022 Nov 29;12:20566. doi: 10.1038/s41598-022-24665-w (PMC9709162; doi:10.1038/s41598-022-24665-w)
Supplement: Supplementary file 2 — Supplementary Information 2. [file 41598_2022_24665_MOESM2_ESM.zip › RAL Raw data-Ñ[▒K/108/HS_ñw╝╨░O▒Kñσ.pdf]

正式摘要(電子簽章：000000225964751)
W52 / W52 ☐ 英文標題(注意:無英文姓名-請補病人基本檔!) ☐【讀取】憑證

臺中榮民總醫院
出院病歷摘要

轉入號： 轉出號：

[redacted]

(8)流水編號	
(9)入院日期	110/08/24 (2021) 直腸外科 W52 - 172
(10)轉科日期	
(11)出院日期	110/09/28 (2021) 住院天數計 35 日

診斷

- 入院：1. Colon Cancer, cT3N1M0, Stage IIIB
  [PHx]
  1. Appendectomy in 1969
  2. HCV s/p medication treatment
- 出院：1. Sigmoid colon adenocarcinoma, cT3N1M0, Stage IIIB, s/p robotic anterior resection on 2021/08/25,
  pT3N1b(2/19, mesocolic LN metastasis), pMMR, MD, AI-, PI-, TuDp-, DisMg 4.5cm-, CRM 4cm-, ExNd-, stage IIIB
  with postoperative leak, s/p Hartmann's procedure and end D-colonostomy on 2021/09/01,
  wound culture: KP, Morganella morganii, E.coli, and Proteus vulgaris. Pus culture: CRAB, KP
  -s/p Tazocin(2021/09/01-09/15), Cefoxitin(09/16-09/28)
  [PHx]
  1. Appendectomy in 1969
  2. HCV s/p medication treatment

主訴

- Bloody stool for 1 month

病史

- This 71 y/o male patient had history of
  1. Appendectomy on 1969
  2. HCV s/p medication treatment

  He had bloody tinged stool on 2021/08/09, and visited our hospital for help. He had colonoscopy on 2021/08/12 and revealed sigmoid colon tumor, 20cm FAV, cT3N1M0, Stage IIIB
  He was admitted for robotic surgery this time.

體檢發現

-
  - General appearance :
    - easy looking , conscious clear , without cardiopulmonary distress
    - Vital signs自動帶入date 體溫(BT) 脈搏(P) 呼吸(R) 血壓(BP) 身高(BH) 體重(BW) BMI
      20210824 36.9℃ 63次/分 19次/分 179/92mmHg 166.9cm 67.5kg 24.2
    - Vital signs: B.P. 179/92 mmHg , P.R. 63 /min , R.R. 19 /min , B.T. 36.9 ℃
  - HEENT :
    - Eyes: no anemic conjunctiva, no icteric sclera, normal EOM
    - ENT: no otorrhea, no rhinorrhea, no erythematous mucosa, no injected tonsil, no deformity
  - Neck: supple, freely movable, trachea midline,no deformity, no engorged jugular vein , no palpable thyroid or lymph node enlargement
  - Chest:
    - Inspection: Grossly normal, symmetric and fully expansion, no accessory muscle used, no operation scar, no spider angiomata, no gynecomastia
    - Palpation: no palpable mass or nodule, no subcutaneous emphysema, no tenderness
    - Ausculation: clear breathing sound
  - Heart:
    - Palpation: no thrill, no heave
    - Percussion: normal heart size
    - Ausculation: regular heart beat, normal S1, S2, no gallop, no murmur
  - Extremities: No deformity, full ranged of motion without limitation, no clubbing finger or nail cyanosis, no pitting edema, no ulcer or pigmentation, no obvious skin lesion, no varicose vein, no bone, joint or muscle tenderness

  CRS CONDITION
  - Karnofsky performance status: (100) Normal, no complaints, no evidence of disease
  - PE of Abdomen:
  - CEA: 3.85 ng/ml
  - Imaging Studies:
  - Colonoscope report: Colonscopy was up to S-colon. Tumor was noted at 20cm FAV, S/p biopsy & tatto was done.
  - Sono , CT of abdomen and chest Films:> Sigmoidal Colon Ca. for lung survey.
    > Several small lung nodules in right upper lobe(axial:14-16, 28), along right major fissure(axial:36, 39) and right lower lobe(axial:41), lung metastasis cannot be totally R/O. Suggest further evaluation or closely F/U.
    > Some small LNs in middle mediastinum.
    > Two hepatic cysts at left hepatic lobe of liver, condition stable.
    > No obvious focal lesion over visible liver, spleen and bil. adrenal glands.
    > DJD change of T-L spine with spur formation.
  - Figures:
    1. colon & rectum

手術日期及方法

- 手術日期及方法20210901 1. Hartmann's procedure(end D-colostomy+ lower rectal stump).
  2. TA (60-4.8, X1) were applied for colon resection.
  3. End D-colostomy was created over the LLQ area
  4. Two 12 Fr. penrose drainage tube was inserted from RLQ site and it was placed over the pelvic area
  5. the fascia was also closed with retension Nylon
  20210825 Robot Assisted Colectomy(L)

住院治療經過

- After admission, the patient received robotic anterior resection on 2021/08/25. After operation, we gradually removed NG and started to try diet. However, he presented with persist abdominal distention, with ascites and air leakage from OP wound. Abdominal CT on 9/1 disclosed pneumoperitoneum with increased omental and mesenteric stranding, loculated ascites. Anastomosis leak was suspected. We therefore performed explore laparotomy on the same day. Previous anastomosis site leak was found, we performed Hartmann's procedure and end D-colostomy. Wound culture yielded KP, Morganella morganii, E.coli, and Proteus vulgaris, pus culture yielded KP and CRAB, s/p Tazocin(2021/09/01-09/15), Cefoxitin(09/16-09/28). OP wound healed well with gradually resolving abdominal distention. Therefore, we gradually removed NG and started try diet. He presented with well digestion and defecation condition. Under relative stable condition, he was discharged. OPD follow up was arranged.

合併症

一般檢查

- 累積報告：

DATE	TIME	ALB	TP	BIL.T	BIL.D	ALKP	AST	ALT	LDH	AMY	LIPASE	NH3	Ferritin	UA
1100824	1732	4.3		0.56	0.13	65	29	20						
1100825	1427	3.6		0.6	0.2		20	15						
1100831	1046	3.0		0.58	0.21	79	14	8						
1100901	2056			1.12				17						

1100902	0856	2.6		1.20	0.53		17	12						
1100903	1007	3.0		0.64	0.28		15	10						
1100906	0924	3.2		0.85	0.38		25	22						
1100909	0855	3.4		1.21				24						
1100913	0859	3.5		0.59	0.25			17						
1100916	0901	3.5		0.4				18						
1100920	0853			0.5				14						
1100923	0845	3.6		0.55				15						
1100927	0949			0.6				14						

DATE	TIME	WBC	RBC	HGB	HCT	MCV	PLT	BLST	NEUT	BAND	SEG	LYM	MON	EOS	BAS
1100824	1731	8030		13.7	40.7		278		71.8			18.4	8.1	1.2	0.5
1100825	1431	13530		13.0	36.9		246		96.0			2.1	1.8	0.0	0.1
1100831	1048	9310		10.7	30.8		235			1.0	91.0	4.0	4.0	0.0	0.0
1100901	2056	12000		12.1	35.6		241			1.0	86.0	2.0	11.0	0.0	0.0
1100902	0852	13370	4.16	12.8	36.2	87.0	271			2.0	80.0	6.0	11.0	0.0	0.0
1100903	1011	13890		11.1			253				86.0	4.0	8.0	0.0	0.0
1100906	0913	21000		12.3			411			1.0	79.0	4.0	10.0	1.0	1.0
1100909	0852	17210		11.6			670			2.0	79.0	6.0	4.0	2.0	1.0
1100913	0954	14710		11.6			768				81.0	10.0	5.0	2.0	1.0
1100916	0912	10710		11.7			579				72.0	10.0	14.0	3.0	1.0
1100920	0933	7920		11.9			448				73.0	14.0	9.0	4.0	0.0
1100923	0847	8010		11.7			388		70.9			16.5	8.0	3.4	1.2
1100927	0947	8810		11.6			352		72.5			15.2	8.5	3.1	0.7

DATE	TIME	NA	K	CL	BUN	CREAT	eGFR	CA	Mg	P	CRP	ESR	PCT	D-Dimer	LACTATE
1100825	1427	136	3.3		11	0.90	88.41	8.3							
1100831	1036														7.5
1100831	1046	135	3.2		24	1.17	65.32	8.5							
1100901	2056	135	3.3	98	19	1.08	71.64	7.4							8.2
1100902	0856	136	3.9		16	1.25	60.34	7.6	2.1	2.5					
1100903	0956														7.1
1100903	1007	136	3.8		16	1.00	78.07	7.8							
1100906	0924	132	4.0		10	0.82	98.16	7.4							
1100909	0855	129	4.2		13	0.85	94.17	7.8							
1100913	0859	132	4.7		15	0.75	108.81	8.4							
1100916	0901	133	4.7		17	0.80	101	8.7							
1100920	0853	132	4.6		19	0.81	99.56	8.9							
1100923	0845	135	3.9		21	0.81	99.56	8.8							
1100927	0949	135	4.0		14	0.77	105.55	9.4							

DATE	TIME	GLUCOSE	HbA1C	CHOL	TG	HDL	LDL	KETONE	CK	CKMB	TRO-T	PT-P	APTT-P	NT-pro BNP
1100902	0856			97	81									
1100903	1016	182												

DATE	TIME	SP-GR	PH	PROT	GLUC	KETON	BILI	UROB	OB	NITR	LEU	RBC	WBC	EPCEL
1100901	1058	1.018	5.5	Trace	Negative	Negative	1+	1.0	Trace	Negative	Negative	2-5	0-2	0-1

DATE	TIME	PH	PCO2	PO2	SO2	BEB	HCO3	TCO2	HGB	FIO2
1100901	2056	7.432	39.9	73.6	95.1	1.7	26.0	27.2		

DATE	TIME	PH	BEB	SCVO2	PO2	HCO3	HGB	PCO2	TCO2
1100903	0959	7.399	-0.9	98.1	107.2	23.7		39.3	24.9

- 放射線報告 :檢驗項目 **:CT ABDOMEN-SURVEY** 報告日期 **:1100901**

主　訴：
Result:Y
Finding:
CT scan of abdomen with IV contrast medium administration and oral contrast medium ingestion:(S-colon cancer with mesocolic lymphadenopathy S/P OP)
>S/P Robot-assisted left colectomy(2021/8/25)
>Pneumoperitoneum with increased omental & mesenteric strandings is mentioned with loculated ascites at right subphrenic space, right paracolic gutter and left mesentery. Recommend clinical correlation and regular follow up.
>Emphysema at visible right chest wall and bilateral anterior abdominal wall, right inguinal space and scrotum
>Mild bilateral pleural effusion is mentioned with subpleural atelectasis in visible RLL and LLL.
>A small calcification(0.3cm) in right lobe of liver
>A small cyst(0.8cm) in left lobe of liver
>DJD change with marginal spur formation of visible T-L spine
>Scoliosis of L-spine with leftward convexity
>Atherosclerotic calcified plaque formation of visible abdominal aorta and bilateral iliac arteries
>No definite evidence of significant focal lesion can be identified in the pancreas, spleen and bilateral kidneys.
Impression:
As above descriptions

檢驗項目 **:CT, LUNG** 報告日期 **:1100823**
主　訴：
Result:Y
Finding:
CT of chest and mediastinum with IV contrast enhancement:
> Sigmoidal Colon Ca. for lung survey.
> Several small lung nodules in right upper lobe(axial:14-16, 28), along right major fissure(axial:36, 39) and right lower lobe(axial:41), lung metastasis cannot be totally R/O. Suggest further evaluation or closely F/U.
> Some small LNs in middle mediastinum.
> Two hepatic cysts at left hepatic lobe of liver, condition stable.
> No obvious focal lesion over visible liver, spleen and bil. adrenal glands.
> DJD change of T-L spine with spur formation.
Impression:
Several nodules in right lung, lung metastasis cannot be totally R/O. Suggest further evaluation or closely F/U.

檢驗項目 **:CT ABDOMEN-SURVEY** 報告日期 **:1100813**
主　訴：
Result:Y
Finding:
Imaging Report Form for Colorectal Carcinoma (AJCC 8th)
1. Imaging Date: 20210812
Imaging Modality, Imaging by
CT scan
Imaging Protocol: CT scan of abdomen with IV and oral contrast enhancement:
2. Tumor location / Size
Location:
Sigmoid
Size:
Measurable: 3.7 cm (largest diameter)
3. Tumor invasion
Tumor invades non-peritonealized pericolonic or perirectal tissues
4. Regional nodal metastasis
No
5. Distant metastasis (In this study)
No or Equivocal
6. Other findings: 1. Asymmetrical wall thickening about 3.7cm in length at S-colon with stranding of pericolic fat plane & small pericolic lymph nodes, R/I malignancy. Please correlate with colonoscopy..
2. Small low attenuation lesions at left lobes of liver, R/O hepatic cysts. Please correlate with abdominal US.

- 3. A tiny calcification at S7 of liver.
  4. gallbladder, pancreas, both kidneys, spleen are without specific abnormality.
  5. No obvious para-aortic or retrocaval lymphadenopathy is noted.
  6. A tiny calcification about 2mm in size at right major fissure(ax.5)
  7. A tiny subpleural nodule about 1mm in size at RLL(Ax.7)
  Impression:
  1. Asymmetrical wall thickening at S-colon, R/I malignancy. Please correlate with colonoscopy.
  2. Other findings, as above descriptio

特殊檢查

病理報告

- 活體組織切片病理報告 手術日期：**110/08/25** 病理號：**S1026447**

  診斷醫師:陳韻安 報告日期:110/08/31
  Pathologic diagnosis:
  1. Sigmoid colon, robotic anterior resection -— Adenocarcinoma, moderately differentiated. Tumor invaded to mesocolic soft tissue and metastasized to mesocolic lymph nodes (2/14). The bilateral cut ends are free of tumor.
  2. IMA root, dissection —- Unremarkable fibroadipose tissue, vessels and lymph nodes (0/2).
  3. IMV root, dissection —- Unremarkable fibroadipose tissue, vessels and lymph nodes (0/3).
  4. Colon, cut end, distal, resection —- Unremarkable.
  Ancillary study for diagnosis:
  IHC stains for PMS2 and MSH6 are done on section C. All above markers are preserved.
  Prognostic and predictive factor:
  1. Tumor size: 3.5x3 cm.
  2. Depth of invasion: Pericolorectal tissue.
  3. Histologic grade: G2: Moderately differentiated.
  4. Angiolymphatic invasion: Not identified.
  5. Perineural invasion: Present.
  6. Tumor deposits (discontinuous extramural extension): Not identified.
  7. Macroscopic tumor perforation: Not identified.
  8. Surgical Margins: a) Proximal Margin: Uninvolved by invasive carcinoma, distance of tumor from margin: 4.5 cm. b) Distal Margin: Uninvolved by invasive carcinoma, distance of tumor from margin: 4.5 cm. c) Radial or Mesenteric Margin: Uninvolved by invasive carcinoma, distance of tumor from margin: 4 cm.
  9. Lymph node status: Involved (2/19).
  10. Extranodal involvement: Not identified.
  11. Tumor regression grading S/P CCRT: No known presurgical therapy.
  12. Type of polyp in which invasive carcinoma arose: Not identified.
  13. Additional pathologic findings: Not identified.
  14. Pathological TNM Stage: pT3N1b(According to the eighth edition, American Joint Committee on Cancer Staging Guidelines for Tumors).
  15. TNM descriptors: Not applicable.
  Gross description:
  The specimen consists of 1) a segment of colon, 13.5 cm in length and 5 cm in proximal cut end circumference, 5 cm in distal circumference, and mesocolic soft tissue. A gray tan firm annular elevated and centrally ulcerative tumor, 3.5x3 cm, is noted, 4.5 cm to the nearest cut end. On cross section, the tumor invades to the mesocolic soft tissue with 4 cm to the radial margin. The serosa is close to the tumor, and there is serosal retraction. The both cut ends are free of tumor grossly. 2) IMA root, a piece of tan yellow soft tissue, 2.5x2x1 cm. 3) IMV root, a piece of yellow tan soft tissue, 4.5x3x1 cm. 4) distal cut end, a piece of tan mucosa-covered soft tissue, 2x2x1.5 cm. Representative part for section: A) proximal cut end B) distal cut end C-E) tumor F) radial margin G) non-tumor mucosa. All for section: H,J-K,L) sp.2,3,4.
  Note:
  1. The interpretation of MMR panel and recommended studies are as below: (1) No loss of nuclear expression of MMR proteins: low probability of microsatellite instability-high (MSI-H) . (2) Loss of nuclear expression of MLH1 and PMS2: testing for methylation of the MLH1 promoter and/or mutation of BRAF is indicated (the presence of a BRAF V600E mutation and/or MLH1 methylation suggests that the tumor is sporadic and germline evaluation is probably not indicated; absence of both MLH1 methylation and of BRAF V600E mutation suggests the possibility of Lynch syndrome, and sequencing and/or large deletion/duplication testing of germline MLH1 may be indicated). (3) Loss of nuclear expression of MSH2 and MSH6: high probability of Lynch syndrome (sequencing and/or large deletion/duplication testing of germline MSH2 may be indicated, and, if negative, sequencing and/or large deletion/duplication testing of germline MSH6 may be indicated). (4) Loss of nuclear expression of MSH6 only: high probability of Lynch syndrome (sequencing and/or large deletion/duplication testing of germline MSH6 may be indicated). (5) Loss of nuclear expression of PMS2 only: high probability of Lynch syndrome (sequencing and/or large deletion/duplication testing of germline PMS2 may be indicated). (6) Please note: There are exceptions to the above IHC interpretations. These results should not be considered in isolation, and clinical correlation with genetic counseling is recommended to assess the need for germline testing.
  2. This case has been peer reviewed by two doctors.
  #T-59300_2 #M-81403_2 2 1105 S0000F
  #T-C4560_2 #M-81403_2 2 1105 00000F

其它

- 隔離註記:接觸防護【CRAB(檢)(20210925)】

出院時情況

- 3.改門診治療

出院指示

藥名	劑量	劑量單位	用法	頻次	數量	數量單位	現況	天數	單位
5mg-Primperan tab	1	TAB	PO	QD	7	TAB	TH	7	天
Econ tab 40mg	1	TAB	PO	QID	28	TAB	TH	7	天
Ultracet tab	1	TAB	PO	QIDPRN	28	TAB	TH	7	天
Mag. Oxide tab	2	TAB	PO	TID	42	TAB	TH	7	天
Norvasc tab 5mg	1	TAB	PO	QD	7	TAB	TH	7	天
Xanax tab 0.5mg	1	TAB	PO	TID	21	TAB	TH	7	天
Unasyn TAB 375mg	1	TAB	PO	Q12H	14	TAB	TH	7	天

出院計劃

- 門診日期
  - date on 2021/10/04

主治醫師 2222H 林俊余 / 住院醫師 9739F 趙雅芸

正式摘要(電子簽章：**000000225964751)**

W52 / W52

doctorRank:B
